# 2,3,4,5-Tetra­fluoro­benzoic acid–4,4′-bipyridine (2/1)

**DOI:** 10.1107/S1600536809027445

**Published:** 2009-07-18

**Authors:** Xiaohui Zhu

**Affiliations:** a School of Chemistry and Chemical Engineering, Taishan Medical University, 271016 Taian, Shandong, People’s Republic of China

## Abstract

The asymmetric unit of the title compound, 2C_7_H_2_F_4_O_2_·C_10_H_8_N_2_, contains one mol­ecule of 2,3,4,5-tetra­fluoro­benzoic acid (tfb) and half of a centrosymmetric 4,4′-bipyridine mol­ecule. Inter­molecular O—H⋯N hydrogen bonds link two tfb mol­ecules and one 4,4′-bipyridine mol­ecule into a trimer. Weak inter­molecular C—H⋯F inter­actions assemble these trimers into a three-dimensional network structure.

## Related literature

For complexes with fluorated carboxyl­ates, see: Ma *et al.* (2006 ▶); Gielen *et al.* (1992 ▶).
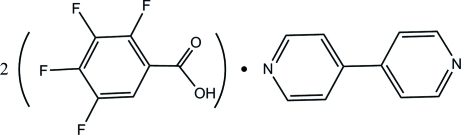


## Experimental

### 

#### Crystal data

2C_7_H_2_F_4_O_2_·C_10_H_8_N_2_
*M* *_r_* = 544.36
Monoclinic, 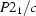

*a* = 6.6517 (7) Å
*b* = 8.3419 (14) Å
*c* = 19.5310 (11) Å
β = 93.181 (2)°
*V* = 1082.1 (2) Å^3^
*Z* = 2
Mo *K*α radiation
μ = 0.16 mm^−1^
*T* = 298 K
0.45 × 0.43 × 0.24 mm

#### Data collection

Bruker SMART APEX diffractometer
Absorption correction: none
5243 measured reflections
1888 independent reflections
1107 reflections with *I* > 2σ(*I*)
*R*_int_ = 0.031

#### Refinement

*R*[*F* ^2^ > 2σ(*F* ^2^)] = 0.037
*wR*(*F* ^2^) = 0.123
*S* = 1.00
1888 reflections
172 parameters
H-atom parameters constrained
Δρ_max_ = 0.22 e Å^−3^
Δρ_min_ = −0.15 e Å^−3^

### 

Data collection: *SMART* (Siemens, 1996 ▶); cell refinement: *SAINT* (Siemens, 1996 ▶); data reduction: *SAINT*; program(s) used to solve structure: *SHELXS97* (Sheldrick, 2008 ▶); program(s) used to refine structure: *SHELXL97* (Sheldrick, 2008 ▶); molecular graphics: *SHELXTL* (Sheldrick, 2008 ▶); software used to prepare material for publication: *SHELXTL*.

## Supplementary Material

Crystal structure: contains datablocks I, global. DOI: 10.1107/S1600536809027445/cv2583sup1.cif

Structure factors: contains datablocks I. DOI: 10.1107/S1600536809027445/cv2583Isup2.hkl

Additional supplementary materials:  crystallographic information; 3D view; checkCIF report

## Figures and Tables

**Table 1:** Hydrogen-bond geometry (Å, °)

*D*—H⋯*A*	*D*—H	H⋯*A*	*D*⋯*A*	*D*—H⋯*A*
C12—H12⋯F4^i^	0.93	2.39	3.105 (3)	134
C8—H8⋯F3^ii^	0.93	2.56	3.308 (3)	138
O1—H1⋯N1^iii^	0.82	1.80	2.620 (2)	174

Symmetry codes: (i) 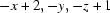; (ii) 
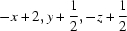
; (iii) 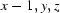.

**Fig. 1.**
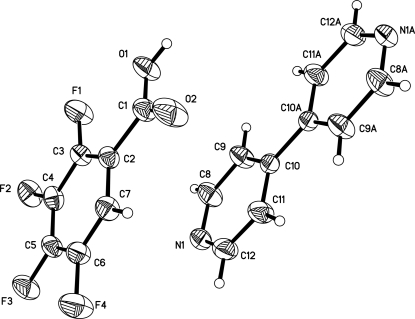
The content of asymmetric unit of the title compound, with atomic numbering and 50% probability displacement ellipsoids [symmetry code: (A) 1-x, 1-y, 1-z].

## supplementary crystallographic information

### Comment

The 2,3,4,5-tetrafluorobenzoic acid has been intensively studied in biological systems and metal complexes which can considerably increase their biological activities because of the presence of fluorine atom (Ma *et al.*, 2006; Gielen *et al.*, 1992). In view of this, we have selected this ligand and acetate cube in the presence of 4,4'-bipyrimidine as co-ligand  to continue the study of fluorated metal compounds. The title compound (Fig. 1)  has been obtained as by-side product.

In the crystal, intermolecular O—H···N hydrogen bonds (Table 1) link two molecules of 2,3,4,5-tetrafluorobenzoic acid and one 4,4'-bipyridine molecule into trimer. Weak intermolecular C—H···F interactions (Table 1) assemble further these trimers into three-dimensional network structure.

### Experimental

All reagents and solvents were used without further purification. This complex was synthesized by the hydrothermal method from a mixture of an methanol solution of 2,3,4,5-tetrafluorobenzoic acid that had been neutralized with sodium hydroxide, 4,4'-bipyrimidine,acetate cube and water in a airtight vessel. The solution was heated at 313 K for 3 d. After reaction, the vessel was cooled slowly down to room temperature to give transparent brown crystals. The block-like crystals were collected and washed with distilled methanol and dried in air (78% yield). Elemental analysis-found:*C*,52.88%,2.27%,5.36%; calc.for C_12_H_6_F_4_NO_2_: C,52.95%,H,2.22%,*N*,5.15%. The elemental analyses were performed with PERKIN ELMER MODEL 2400 SERIES II.

### Crystal data

**Table:**

2C_7_H_2_F_4_O_2_·C_10_H_8_N_2_	*F*(000) = 548
*M**_r_* = 544.36	*D*_x_ = 1.671 Mg m^−^^3^
Monoclinic, *P*2_1_/*c*	Mo *K*α radiation, λ = 0.71073 Å
*a* = 6.6517 (7) Å	Cell parameters from 1438 reflections
*b* = 8.3419 (14) Å	θ = 3.2–23.9°
*c* = 19.5310 (11) Å	µ = 0.16 mm^−^^1^
β = 93.181 (2)°	*T* = 298 K
*V* = 1082.1 (2) Å^3^	Block, colourless
*Z* = 2	0.45 × 0.43 × 0.24 mm

### Data collection

**Table:**

Bruker SMART APEX diffractometer	1107 reflections with *I* > 2σ(*I*)
Radiation source: fine-focus sealed tube	*R*_int_ = 0.031
graphite	θ_max_ = 25.0°, θ_min_ = 2.1°
φ and ω scans	*h* = −6→7
5243 measured reflections	*k* = −7→9
1888 independent reflections	*l* = −22→23

### Refinement

**Table:**

Refinement on *F*^2^	Primary atom site location: structure-invariant direct methods
Least-squares matrix: full	Secondary atom site location: difference Fourier map
*R*[*F*^2^ > 2σ(*F*^2^)] = 0.037	Hydrogen site location: inferred from neighbouring sites
*wR*(*F*^2^) = 0.123	H-atom parameters constrained
*S* = 1.00	*w* = 1/[σ^2^(*F*_o_^2^) + (0.062*P*)^2^ + 0.1216*P*] where *P* = (*F*_o_^2^ + 2*F*_c_^2^)/3
1888 reflections	(Δ/σ)_max_ < 0.001
172 parameters	Δρ_max_ = 0.22 e Å^−^^3^
0 restraints	Δρ_min_ = −0.15 e Å^−^^3^

### Special details

**Table:**

Geometry. All e.s.d.'s (except the e.s.d. in the dihedral angle between two l.s. planes) are estimated using the full covariance matrix. The cell e.s.d.'s are taken into account individually in the estimation of e.s.d.'s in distances, angles and torsion angles; correlations between e.s.d.'s in cell parameters are only used when they are defined by crystal symmetry. An approximate (isotropic) treatment of cell e.s.d.'s is used for estimating e.s.d.'s involving l.s. planes.
Refinement. Refinement of *F*^2^ against ALL reflections. The weighted *R*-factor *wR* and goodness of fit *S* are based on *F*^2^, conventional *R*-factors *R* are based on *F*, with *F* set to zero for negative *F*^2^. The threshold expression of *F*^2^ > σ(*F*^2^) is used only for calculating *R*-factors(gt) *etc*. and is not relevant to the choice of reflections for refinement. *R*-factors based on *F*^2^ are statistically about twice as large as those based on *F*, and *R*- factors based on ALL data will be even larger.

### Fractional atomic coordinates and isotropic or equivalent isotropic displacement parameters (Å^2^)

**Table:**

	*x*	*y*	*z*	*U*_iso_*/*U*_eq_	
F2	0.7895 (2)	0.12908 (19)	0.19446 (8)	0.0718 (5)	
C2	0.5194 (3)	0.1029 (3)	0.34972 (12)	0.0468 (6)	
C3	0.5693 (3)	0.1433 (3)	0.28441 (12)	0.0489 (6)	
O1	0.2294 (3)	0.2650 (2)	0.34704 (9)	0.0694 (6)	
H1	0.1322	0.2933	0.3681	0.104*	
C10	0.5879 (3)	0.4735 (3)	0.48110 (12)	0.0470 (6)	
F3	1.0440 (2)	−0.05674 (19)	0.27137 (8)	0.0749 (5)	
C7	0.6499 (4)	0.0052 (3)	0.38789 (13)	0.0534 (7)	
H7	0.6191	−0.0250	0.4319	0.064*	
F1	0.4516 (2)	0.2331 (2)	0.24231 (8)	0.0806 (5)	
C5	0.8729 (3)	−0.0054 (3)	0.29677 (13)	0.0508 (6)	
N1	0.9175 (3)	0.3732 (3)	0.40908 (11)	0.0588 (6)	
C4	0.7454 (4)	0.0890 (3)	0.25813 (13)	0.0517 (7)	
C6	0.8238 (4)	−0.0474 (3)	0.36163 (13)	0.0553 (7)	
C1	0.3355 (4)	0.1634 (3)	0.38353 (15)	0.0559 (7)	
O2	0.2975 (3)	0.1188 (3)	0.43978 (11)	0.0916 (7)	
F4	0.9521 (2)	−0.1423 (2)	0.39827 (9)	0.0884 (6)	
C9	0.6276 (4)	0.5363 (3)	0.41783 (14)	0.0641 (8)	
H9	0.5431	0.6140	0.3978	0.077*	
C8	0.7922 (4)	0.4839 (4)	0.38453 (15)	0.0683 (8)	
H8	0.8163	0.5293	0.3423	0.082*	
C11	0.7186 (4)	0.3584 (4)	0.50629 (14)	0.0705 (8)	
H11	0.6988	0.3110	0.5485	0.085*	
C12	0.8799 (4)	0.3124 (4)	0.46918 (15)	0.0723 (8)	
H12	0.9665	0.2343	0.4877	0.087*	

### Atomic displacement parameters (Å^2^)

**Table:**

	*U*^11^	*U*^22^	*U*^33^	*U*^12^	*U*^13^	*U*^23^
F2	0.0840 (11)	0.0793 (10)	0.0551 (9)	0.0070 (8)	0.0311 (8)	0.0059 (8)
C2	0.0446 (13)	0.0492 (14)	0.0477 (14)	−0.0005 (11)	0.0116 (11)	−0.0041 (11)
C3	0.0489 (14)	0.0480 (14)	0.0505 (15)	0.0057 (11)	0.0087 (12)	0.0008 (12)
O1	0.0577 (11)	0.0810 (13)	0.0721 (13)	0.0203 (10)	0.0250 (9)	0.0034 (11)
C10	0.0375 (12)	0.0598 (15)	0.0440 (13)	−0.0011 (11)	0.0045 (10)	−0.0094 (12)
F3	0.0574 (9)	0.0898 (12)	0.0803 (11)	0.0176 (8)	0.0280 (8)	−0.0038 (9)
C7	0.0533 (15)	0.0590 (16)	0.0492 (15)	0.0012 (13)	0.0143 (12)	0.0037 (12)
F1	0.0824 (11)	0.0976 (13)	0.0633 (11)	0.0354 (10)	0.0166 (8)	0.0160 (9)
C5	0.0439 (14)	0.0535 (16)	0.0564 (15)	0.0039 (12)	0.0158 (12)	−0.0068 (12)
N1	0.0457 (12)	0.0711 (15)	0.0604 (15)	0.0057 (11)	0.0119 (10)	−0.0101 (12)
C4	0.0582 (15)	0.0520 (15)	0.0467 (15)	−0.0042 (12)	0.0208 (13)	−0.0020 (12)
C6	0.0542 (15)	0.0574 (16)	0.0546 (16)	0.0096 (13)	0.0045 (13)	0.0031 (13)
C1	0.0462 (14)	0.0623 (17)	0.0605 (18)	0.0011 (13)	0.0154 (13)	−0.0058 (14)
O2	0.0743 (13)	0.1325 (18)	0.0718 (14)	0.0290 (13)	0.0381 (11)	0.0212 (13)
F4	0.0800 (11)	0.1119 (14)	0.0742 (12)	0.0383 (10)	0.0110 (9)	0.0204 (10)
C9	0.0581 (16)	0.0693 (18)	0.0671 (18)	0.0151 (14)	0.0220 (14)	0.0075 (15)
C8	0.0649 (18)	0.0769 (19)	0.0662 (18)	0.0088 (16)	0.0311 (15)	0.0075 (16)
C11	0.0585 (16)	0.107 (2)	0.0469 (16)	0.0271 (17)	0.0120 (12)	0.0057 (15)
C12	0.0583 (17)	0.103 (2)	0.0563 (18)	0.0269 (16)	0.0065 (14)	−0.0013 (17)

### Geometric parameters (Å, °)

**Table:**

F2—C4	1.336 (3)	C7—H7	0.9300
C2—C3	1.378 (3)	C5—C4	1.356 (3)
C2—C7	1.379 (3)	C5—C6	1.371 (3)
C2—C1	1.509 (3)	N1—C12	1.315 (3)
C3—F1	1.334 (3)	N1—C8	1.317 (3)
C3—C4	1.381 (3)	C6—F4	1.341 (3)
O1—C1	1.292 (3)	C1—O2	1.200 (3)
O1—H1	0.8200	C9—C8	1.375 (3)
C10—C11	1.368 (3)	C9—H9	0.9300
C10—C9	1.381 (4)	C8—H8	0.9300
C10—C10^i^	1.484 (4)	C11—C12	1.382 (3)
F3—C5	1.337 (2)	C11—H11	0.9300
C7—C6	1.364 (3)	C12—H12	0.9300
			
C3—C2—C7	117.9 (2)	C5—C4—C3	120.1 (2)
C3—C2—C1	124.5 (2)	F4—C6—C7	121.1 (2)
C7—C2—C1	117.5 (2)	F4—C6—C5	117.8 (2)
F1—C3—C2	123.0 (2)	C7—C6—C5	121.0 (2)
F1—C3—C4	115.9 (2)	O2—C1—O1	124.9 (2)
C2—C3—C4	121.1 (2)	O2—C1—C2	121.0 (3)
C1—O1—H1	109.5	O1—C1—C2	114.2 (2)
C11—C10—C9	116.0 (2)	C8—C9—C10	119.9 (3)
C11—C10—C10^i^	122.2 (3)	C8—C9—H9	120.0
C9—C10—C10^i^	121.8 (3)	C10—C9—H9	120.0
C6—C7—C2	120.6 (2)	N1—C8—C9	123.7 (3)
C6—C7—H7	119.7	N1—C8—H8	118.1
C2—C7—H7	119.7	C9—C8—H8	118.1
F3—C5—C4	119.9 (2)	C10—C11—C12	120.2 (3)
F3—C5—C6	120.8 (2)	C10—C11—H11	119.9
C4—C5—C6	119.3 (2)	C12—C11—H11	119.9
C12—N1—C8	116.6 (2)	N1—C12—C11	123.6 (3)
F2—C4—C5	120.0 (2)	N1—C12—H12	118.2
F2—C4—C3	119.9 (2)	C11—C12—H12	118.2
			
C7—C2—C3—F1	178.0 (2)	F3—C5—C6—F4	−0.5 (4)
C1—C2—C3—F1	−4.4 (4)	C4—C5—C6—F4	179.3 (2)
C7—C2—C3—C4	−0.6 (4)	F3—C5—C6—C7	179.7 (2)
C1—C2—C3—C4	176.9 (2)	C4—C5—C6—C7	−0.5 (4)
C3—C2—C7—C6	0.8 (4)	C3—C2—C1—O2	177.4 (3)
C1—C2—C7—C6	−176.9 (2)	C7—C2—C1—O2	−5.1 (4)
F3—C5—C4—F2	0.8 (4)	C3—C2—C1—O1	−3.2 (4)
C6—C5—C4—F2	−179.0 (2)	C7—C2—C1—O1	174.3 (2)
F3—C5—C4—C3	−179.5 (2)	C11—C10—C9—C8	−0.8 (4)
C6—C5—C4—C3	0.7 (4)	C10^i^—C10—C9—C8	179.9 (3)
F1—C3—C4—F2	0.8 (3)	C12—N1—C8—C9	−0.5 (4)
C2—C3—C4—F2	179.6 (2)	C10—C9—C8—N1	0.8 (5)
F1—C3—C4—C5	−178.9 (2)	C9—C10—C11—C12	0.6 (4)
C2—C3—C4—C5	−0.1 (4)	C10^i^—C10—C11—C12	179.8 (3)
C2—C7—C6—F4	179.9 (2)	C8—N1—C12—C11	0.2 (4)
C2—C7—C6—C5	−0.3 (4)	C10—C11—C12—N1	−0.3 (5)

Symmetry codes: (i) −*x*+1, −*y*+1, −*z*+1.

### Hydrogen-bond geometry (Å, °)

**Table:**

*D*—H···*A*	*D*—H	H···*A*	*D*···*A*	*D*—H···*A*
C12—H12···F4^ii^	0.93	2.39	3.105 (3)	134
C8—H8···F3^iii^	0.93	2.56	3.308 (3)	138
O1—H1···N1^iv^	0.82	1.80	2.620 (2)	174

Symmetry codes: (ii) −*x*+2, −*y*, −*z*+1; (iii) −*x*+2, *y*+1/2, −*z*+1/2; (iv) *x*−1, *y*, *z*.
